# The Dublin Practice of Midwifery

**Published:** 1834-10-01

**Authors:** 


*The Dublin Practice of Midwifery.* By HENRY MAUNSELL, M.D., Member of the Royal College of Surgeons in Ireland, Superintending Accoucheur to the Wellesley Female Institution, &c.—London, 1834. 18mo. pp. 244.

THIS is, upon the whole, a useful little work, containing a summary of the principal practical points connected with the art of midwifery; and, although it may reasonably be doubted whether such a book was really wanted, yet, as it is usual for every teacher, or nearly so, to have his own Manual, we see no reason why this privilege should be denied to Dr. Maunsell. Much novelty ought not to be expected in such productions, their chief use being that of a text-book, or syllabus of the lectures, which is of great service to the pupil during his attendance on the course, as, by a careful and attentive perusal, he is enabled to recall to his memory the topics discussed by the lecturer. We do not apprehend that the "Dublin Practice of Midwifery" will be of more service than the other numerous Outlines, Elements, and Manuals, which the press has of late brought forth; and, as a work of reference, it will never be looked at, while the invaluable works of Denman and Burns are in existence: it will be read chiefly by Dr. M.'s pupils; and, for the reasons stated, we would strongly recommend it to them all. We cannot, however, refrain from expressing our surprise that any one with a moderate share of brains,—any one, indeed, belonging to the favoured Caucasian variety of the human species,—should ever ask a question which our author tells us is commonly put to obstetric teachers by students. It is this, "What book do you recommend me to take to the lying-in room?" (*Preface*, p. iii.) The student might as well ask, "What book do you recommend me to take to the rheumatic room, or the dyspeptic house?" We fear that a harsh accoucheur would suspect his diffident pupil to belong to the steno-bregmate or platy-bregmate races, and would recommend him to practise some less difficult art than that of medicine. What would the anxious lady in the straw think of her attendant when she found him conning his obstetrical hornbook?

Our author is necessarily very brief in his description of the anatomy of the parts concerned in the process of parturition, and we think rather more so than he ought to have been in some of his practical remarks: for instance, in his directions for the introduction of the catheter, he has not mentioned the alterations in the position of the bladder, produced by a variety of circumstances, which must, of course, produce a corresponding variation in the situation of the urethra and its orifice. This simple operation may be rendered a very difficult one, if we do not previously acquaint ourselves with these altered situations of the parts. In common cases, however, the following plan will be found a very good one:

"If the operation is to be performed while the woman is in bed, she may lie upon her back, or, what is better, upon her left side, with the hips projecting over the edge of the bed. The left forefinger of the operator is then to be introduced to a short distance (about the length of the first joint) into the vagina, and carried forward to the symphysis pubis. By this measure the urethra will be easily discovered lying between the finger and the pubis. It resembles in feel the corpus spongiosum of the male urethra, but is usually rather thicker. The finger is then to be drawn lightly forward along the urethra, until its tip sinks into the pit marking the orifice, in contact with which it is to be held. The catheter, held loosely between the right thumb and forefinger, is next to be passed along the front of the left forefinger, in a direction somewhat backwards, when it at once slips into the orifice of the urethra. The handle should then be slightly depressed, and the instrument passed on into the bladder; during its introduction the point may catch in some of the mucous lacunæ, upon which it should of course be withdrawn a little, and passed forward with a slight variation of its direction. The urethra is from an inch to two inches long, so that in an ordinary case, where there is no disease, we should expect the urine to flow when the catheter has passed in to the distance of two inches. If it should be necessary, the operation may be performed while the patient sits upon the edge of a chair, the operator kneeling before her, and passing his hand between her thighs. In either case, exposure of the woman's person should be carefully avoided. By adopting the plan just mentioned, instead of that usually directed in books, we shall get rid of the necessity for irritating the clitoris, which, for obvious reasons, is a very considerable improvement." (P. 30.)

We believe many of the difficulties frequently met with by some practitioners would be obviated by bearing in mind the usual position of the gravid uterus. Dr. M. observes,

"The situation of the full-sized womb is oblique, with the os directed backwards towards the sacrum, and the fundus forwards so that its axis is nearly identical with that of the brim, being described by an imaginary right line passing from the scrobiculus cordis to the point of the sacrum, a circumstance that requires to be understood, when it is necessary to pass the hand into the uterus." (P. 47.)

With regard to the arrangement of the muscular fibres of the uterus, it is stated that

"The structures of the uterus continue essentially the same as in the unimpregnated condition, but undergo a remarkable development. The muscular fibres, which appeared so irregular in the virgin womb, now exhibit a definite arrangement into layers. The outermost of these fibres arise from the round ligaments, and, regularly diverging, spread over the fundus until they unite. According to Sir C. Bell, the round ligaments are the tendons of this muscle, and serve as fixed points from which it acts in bringing the womb down into the pelvis at the commencement of labour, and giving its mouth the proper direction. In the substance of the organ, internal to this layer, the muscular fibres have a circular direction near the fundus, and a longitudinal near the cervix: they are, however, interwoven together in a very intricate manner; and when they act fully, must have a very powerful effect in constringing the blood-vessels, which pass betweeen them, and so preventing hemorrhage. Their action, during labour, is to open the os tincæ, and draw it, as it were, over the child's head. The most internal stratum of muscle is arranged with the fibres in two sets of concentric circles, each having the orifice of one of the tubes as its centre. These two muscles, if we may so call them, interweave together at their circumferences, and have proceeding from them, on each side, broad longitudinal bands of fibres, which assist the external muscles in bringing the fundus towards the os, and in drawing the latter over the child's head. The circular portions are supposed to corrugate and diminish in size the internal surface of the uterus, after the child has been expelled, and so draw it off, as it were, from the placenta, which, having no power of diminishing its own area, must, of course, separate from the surface to which it is attached when the latter is diminished in the way mentioned.

"The thickness of the uterine parietes is nearly the same as in the unimpregnated state: in the part to which the placenta is attached, it is perhaps a little thicker. Its substance throughout is more spongy and vascular, and has the sinuses much more developed than before conception." (P. 48.)

We do not agree with the author in his statement that "the thickness of the uterine parietes is nearly the same as in the unimpregnated state." We believe there to be a greatly increased degree of thickness, which remains for many days after delivery, and which we have had frequent opportunities of witnessing. In cases of ruptured uterus, this circumstance may be very satisfactorily demonstrated.

Dr. M. adheres, and we think very properly, to the old and generally received opinion regarding the formation of the *decidua reflexa.*

The utility of pressure over the uterine region, in order to secure permanent contraction after the birth of the child, is justly appreciated by Dr. M., and therefore we would ask him, why defer the application of "the binder" until after delivery? It is a much better plan to place this around the abdomen as soon as the second stage has commenced, and to keep up a gradual and uniform degree of compression, by repeatedly tightening it as it slackens, so that the uterus shall at no time be without this support. We believe the bandage, properly applied and attended to, is useful in the prevention of hemorrhage; and we are quite sure that, where there have been large losses of blood, or even a disposition to them, that it would be unsafe to shift the position of the patient, even in that trifling degree required for the application of the binder. The following recommendation cannot be too strongly pressed upon the attention of the young accoucheur.

"After the delivery has been entirely accomplished, it is always advisable to wait near the patient for at least half an hour; and we should, on no account, leave her then if there be any disposition to hemorrhage, or even an unusual vascular excitement. Before taking our departure, we should always carefully ascertain, first, that the uterus is properly contracted, and the binder well adjusted; secondly, that the pulse is regular; and, thirdly, that there is no danger of hemorrhage from the child's funis." (P. 106.)

We are glad to find the author discountenancing that unwise (might we not almost say unwarrantable?) interference insisted upon by some as requisite for the rectification of irregular positions of the head. We entirely concur in opinion with him, that such a course of proceeding is to be deprecated: these cases are almost invariably brought to a satisfactory conclusion by the efforts of nature alone, although there will necessarily, or at least generally, be a protracted labour.

The remarks on the use of the *Ergot of Rye* are evidently the result of sound practical observation: we would wish some of our senior practitioners to follow the advice given, where it is observed,

"The mode in which I have been in the habit of administering ergot is, to infuse ʒss. of the powder in a tea-cupful of boiling water for fifteen minutes, and then give the whole of the infusion with a third of the infused powder, adding a little milk. If this has no effect, it may be repeated in fifteen minutes; but I think it unadvisable and useless to give a third dose: if the two first produce no pains, another will not have a beneficial action.

"The circumstances which contra-indicate the use of this drug should be accurately understood. It never should be given until the os uteri is completely dilated, nor when there is malformation of the pelvis, or rigidity of the soft parts. If used when the os uteri is undilated, its effect would be similar to, and equally injurious with, too early rupture of the membranes: under the latter circumstances, it might cause lacerations of the uterus, or of the other soft parts. It never should be given when there is any preternatural presentation that may require to be rectified, nor in convulsions, nor when there is any tendency to head symptoms. In the first case, by increasing the uterine action, it would of course increase the difficulties; and in the two last it would be unsafe, for reasons presently to be mentioned.

"On the other hand, if the passages be well prepared and dilated, the os uteri fully open, and the head low down in the pelvis, with plenty of room; in fact, nothing but the want of pains preventing its expulsion, we may safely use ergot in the manner above mentioned.

"It may be supposed that greater success would attend the employment of larger quantities of the medicine; but I am fully persuaded that these cannot be employed without exposing the patient to considerable risk. In several instances, I have observed delirium to follow the exhibition of large doses of ergot: it almost invariably depresses the pulse; and I have known it to produce coma and stertorous breathing, without at all affecting the uterus. If it produce these effects, it is manifestly improper when any head symptoms or tendency to them exist." (P. 118.)

We thought that the use of the crotchet after perforation of the head had become nearly obsolete, and we were therefore rather surprised at the author's statement, that he had always found "it answer his purpose, without ever doing mischief." We are not disposed to question the *possibility* of its being used by an experienced hand with tolerable safety; but Dr. Maunsell should recollect he is writing for his juniors, and surely he would not place a crotchet in the hands of his pupils, in preference to that powerful, yet safe instrument, invented by Mr. Holmes. We know not why, but there seems to be a great disinclination on the part of the profession to do justice to this gentleman: we candidly confess ourselves under great obligations to him for the invention of his craniotomy forceps. An author, who well knew human nature, has declared that,

"Convince a man against his will,
He's of the same opinion still."

Such, however, is not our case: we acknowledge we *were* convinced somewhat against our will, having been unwisely prejudiced against the instrument; but bedside experience (the best in such cases,) has taught us that, in cases of great disproportion, there is nothing, by whatever name it may be known, that will so effectually answer the purpose.

Some very practical though concise directions are given for the management of *flooding*, but we see nothing novel in them. The author, in speaking of *turning*, in placental presentations, well observes,

"In performing the operation, we introduce the hand, as slowly as we please, in the manner and with the precautions already laid down, until it reaches the os uteri; it must then either be passed through the placenta, or by the side of it, into the cavity. During this step, the operator has need of all his coolness and resolution. There is usually a frightful increase of hemorrhage, the blood gushing in torrents along his arm; if, panic-stricken, he withdraws for a moment, the woman is inevitably lost; but by pushing on boldly and steadily, he soon brings his wrist and arm, as an effectual plug, into the mouth of the womb, and the discharge immediately ceases. There is then time for consideration, and the feet must be deliberately sought for and brought down in the usual way into the vagina. There will seldom be any more hemorrhage, and the rest of the delivery is to be accomplished as in a footling case. The placenta will generally be found loose in the vagina: I have sometimes found it to come along with my hand and the child's feet out of the vulva. Great attention is necessary to secure subsequent contraction of the uterus, and if the woman require it she should be supported with stimulants during the operation." (P. 169.)

Our author is perfectly right in avoiding the use of opium in flooding cases: he says,

"In the treatment of all forms of hemorrhage, large doses of opium have been very much recommended. Excepting in small quantities, as a stimulant, when collapse was to be dreaded, I have not been in the habit of using it. It may no doubt be useful in spasmodic contraction, but I apprehend it is rather too much to expect from any drug that it will obey our pleasure, in relaxing the uterus when we wish to turn a child, and in causing it to contract when we desire the expulsion of the placenta." (P. 177.)

No notice is taken of *transfusion*, as a remedy in the worst forms of hemorrhage. This we look upon as a very great omission; for, although there can be no doubt that, where a labour is properly conducted, and the principles of treatment in these cases are early put in practice, the operation is very seldom required, yet we must acknowledge, unless we wilfully shut our eyes to facts, that cases may occur, nay *have*

occurred, in which the patient's life could not have been saved by the ordinary means.

The author has followed the old, and we think not the best plan, by making several species of *puerperal fever*, although he acknowledges that the first variety "differs but little from ordinary peritonitis." We have perused his description, and cannot find even the "little" difference alluded to: it is, in good truth, common inflammation of the peritoneum, and requires the same treatment as at other times. It is almost certainly recovered from, and is not contagious. The second variety differs in its symptoms, in its result, (the patient being generally destroyed by it,) and is capable of being conveyed from one patient to another; and on this account we decidedly object to their being called by the same name.

Dr. Maunsell makes some very sensible remarks on *phlegmasia dolens*. The post-mortem appearances clearly show that this disease consists in a very extended inflammation of the cellular structure.

"The pathology of phlegmasia dolens is still extremely obscure. By the older writers it was supposed to be an irregular deposit of the milk (*depôt du lait*); by others, an extravasation of lymph, in consequence of rupture of the lymphatics; and, by some, a general inflammatory state of the same class of vessels. None of these hypotheses account for the symptoms, nor are they supported by post mortem observations. I cannot avoid expressing the same opinion with respect to Dr. Davis's idea, that the disease is phlebitis of the crural veins. Phlegmasia dolens is well known not to be in general a fatal disease, and it is equally notorious, that phlebitis in any part of the body is particularly mortal. Besides this objection, I cannot see that the phenomena of the disease are at all explainable upon the idea of its being venous inflammation. In the generality of cases of the latter malady, there is nothing corresponding to the peculiar firm swelling of phlegmasia dolens, and an attentive examination of Dr. Lee's cases of actual phlebitis will show that they were very distinguishable from the other disease. I do not mean to deny, however, that inflammation and suppuration of the veins is often to be found upon examination of the bodies of those who have died of phlegmasia dolens, but it appears to me that the evidence already in existence does not prove that this inflammation is the cause of the swelled leg, but merely that it supervenes, in certain cases, upon that disease. The disease appears to me to consist in an inflammation of the cellular tissue, occasioning an effusion of coagulating lymph; but how the inflammation is excited, or why it produces those peculiar effects, has not yet been discovered.

"The prognosis, when the disease is uncomplicated, is favorable, but recovery is always slow and very protracted." (P. 230.)

